# OSdream: An online survival and differential analysis tool of recurrence and metastasis of pan-cancers

**DOI:** 10.1016/j.gendis.2024.101446

**Published:** 2024-10-30

**Authors:** Huimin Li, Qiang Wang, Yunyi Liu, Lin Chen, Qiwei Zhao, Longxiang Xie, Lu Zhang, Zhongyi Yan, Guosen Zhang, Yali Han, Wan Zhu, Xiangqian Guo

**Affiliations:** a Institute of Biomedical Informatics, Cell Signal Transduction Laboratory, Bioinformatics Center, Henan Provincial Engineering Center for Tumor Molecular Medicine, School of Basic Medical Sciences, Henan University, Kaifeng, Henan 475004, China; b Institute of Biomedical Informatics, School of Software, Henan University, Kaifeng, Henan 475004, China; c Department of Anesthesia, Stanford University, Stanford, CA 94305, United States

Recurrence and metastasis are the main causes of cancer-related death and significantly decrease the survival rate of tumor patients. Cancer metastasis can initiate in the very early stage of malignant progression. Unfortunately, cancer patients may not necessarily feel or be diagnosed until metastatic clinical symptoms appear several months or years later.^1^ Metastatic cancer patients have lower five-year survival rates and the tumor often relapses.^2^ Cancer recurrence remains a major troublesome clinical problem which usually leads to treatment failure. Many cancer patients developed *in situ* recurrence or metastatic recurrence within five years after tumor resection.^3^ Accurate prediction of cancer recurrence and metastasis facilitates identifying high-risk patients and guiding appropriate treatment, offering the opportunity to prolong patients' survival.

The initiation and progression of cancer are usually induced by the accumulation of molecular changes, whose analysis would facilitate finding diagnostic or prognostic biomarkers for predicting recurrence and metastasis. Web tools or databases can be developed to screen molecules or genes playing vital roles in tumor metastasis or recurrence. TNMplot was ever constructed for differential analysis among normal, tumor, and metastatic tissues, without survival analysis function.^4^ Recurrence/metastasis-free survival acting as clinical outcomes are significant in analyzing the probability of recurrence and metastasis, screening high-risk patients, and developing therapeutic strategies. Therefore, it is necessary to develop a web tool to analyze the role of genes in recurrence/metastasis-free survival, facilitate the evaluation and screening of prognostic genes for tumor recurrence and metastasis, and further contribute to precise medicine.

In the construction of a tool to effectively predict metastasis and recurrence, we respectively used SQL Server and HTML 5.0 for data storage and developing web interfaces. Java is connected to R by the R package ‘R serve' and used to output analysis results, which include survival analysis and differential analysis (Fig. 1A). For survival analysis, R packages including “ggplot2”, “ggpubr”, “magrittr”, “survminer”, and “survival” were used to perform the outcome analysis and output the Kaplan–Meier plot, R packages “pheatmap”, “ggplot2”, “ggpubr”, and “ggsci” were used in subgroup differential analysis to produce the gene expression heatmap and box plot. About the data, we collected 144 transcriptional profiling datasets with clinical follow-up data of 33 cancer types and 22,919 samples from The Cancer Genome Atlas Gene (TCGA), Expression Omnibus (GEO) databases, and literatures. Of these, 50 expression profiling datasets, composed of 12,722 samples covering 29 tumor types, have RFS (recurrence-free survival) information, while 21 expression profiling datasets, composed of 3505 samples covering 5 tumor types, have MFS (metastasis-free survival) information. Fifty expression profiling datasets with 13,371 samples covering 29 tumor types have primary and recurrent tumor tissues for differential analysis, while 73 expression profiling datasets with 6043 samples covering 21 tumor types have normal tissues and primary and metastasized tumor tissues for differential analysis (Figs. S1A–D; Table S1–3). The homepage provides four application modules that cater to two functions: prognosis analysis and differential analysis. The four application modules are the RFS and the MFS modules for prognosis analysis, the DEGs (differentially expressed genes) of the Recurrence module, and the DEGs of the Metastasis module for differential analysis, and the tool was entitled Online Survival and Differential analysis of Recurrence and Metastasis (OSdream) which is freely available to users at https://bioinfo.henu.edu.cn/OSdream/OSdream.html.

**Figure 1:**
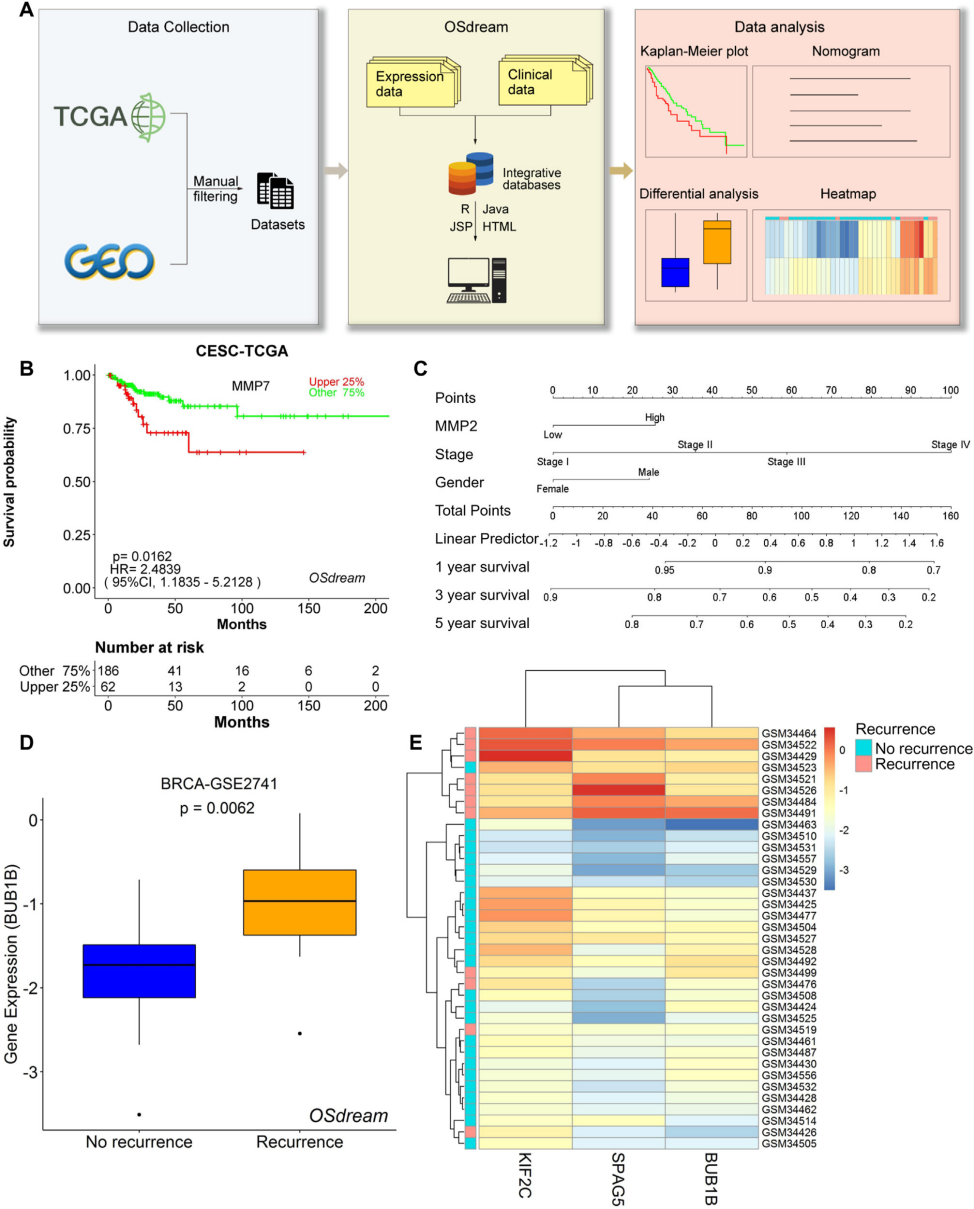
The system flow diagram and the exhibition of outputted results of OSdream. **(A)** System flow diagram of OSdream construction **(B, C)** Kaplan–Meier plot (B) and nomogram (C) for the prognosis analysis. **(D, E)** Box plot (D) and heatmap (E) for the differential analysis.

OSdream is a web tool that evaluates the prognostic and clinical values of genes in tumor recurrence and metastasis in pan-cancers. To measure the prognostic role of genes in tumor recurrence and metastasis, users need to select either the RFS or MFS module based on the query aim, respectively (Fig. S2A, B). To start the RFS/MFS prognosis analysis, users need to enter one official gene symbol, set appropriate analysis parameters including cancer type, data source, cut-off value, and various clinical characters, and then click the “Plot” button, the prognosis analysis of queried gene will be performed and the Kaplan–Meier curve with HR (hazard ratio) will be generated on the output webpage (Fig. 1B). Each probe and the averaged prognostic values for genes will be calculated and outputted in OSdream. A gene may have multiple transcripts due to alternative splicing. As results of alternative splicing or transcribing, different probes are often designed to measure expressional levels of RNA or RNA isoforms and may indicate different or even opposite prognostic values. For example, in the MFS module, in the dataset GSE12276 of breast invasive carcinoma (BRCA), probe 218834_s_at of TMEM132A predicts an adverse prognosis, while probe 232419_at of TMEM132A is related to a favorable prognosis, exhibiting opposite prognostic significance to probe 218834_s_at (Fig. S3A, B). Probe 215165_x_at and 239800_at of UMPS also exhibit opposite prognostic values (Fig. S3C, D). Additionally, the univariate and multivariate Cox regression analyses will be performed, and the analysis results will be presented on the output webpage as well. The nomogram referring to the multivariate Cox analysis results will be built for personalized outcome prediction (Fig. 1C).

To measure the differential expression of genes in samples between patients with recurrence and without recurrence, between patients with metastasis and without metastasis, and between primary tumor and its metastasis, users can select the DEGs of the Recurrence module and the DEGs of the Metastasis module, respectively (Fig. S2A, B). To start the analysis in either of the above two modules, users need to first enter one or multiple official gene symbols in the input box and then click the “Plot” button, the differential analysis for tumor metastasis or recurrence will be performed and the results will be presented as a box plot and a heatmap (Fig. 1D, E). The box plot intuitively and statistically shows differential expression of one or multiple genes in samples between patients with recurrence and without recurrence, between patients with metastasis and without metastasis, and between primary tumor and its metastasis. The heatmap visualizes the co-expression patterns of queried genes across all the tumor samples regarding the processes of recurrence and metastasis. Both the box plot and heatmap assist with the verification and identification of potential markers predicting metastasis or recurrence. To further clarify the predictive power of OSdream, we collected eight biomarkers that were reported or undergoing clinical application for predicting tumor metastasis or recurrence and examined their prediction performance by the four modules in OSdream, and the analysis results based on OSdream showed that the prediction capacity of these biomarkers was consistent with that in prior reports or clinical practice, and the performance test results were exhibited in Table S4 and Figure S4A–F, 5A–F, 6A–F, 7A–F. The above validations indicated that OSdream may be a reliable tool to assist in predicting tumor recurrence and metastasis.

The clinical implementation of effective biomarkers for tumor recurrence or metastasis can facilitate the early diagnosis and stratification of tumor patients with distinct risks. So far, only a limited number of them have been translated into clinical use, such as the clinical application of Oncotype Dx^5^, MammaPrint, and Prosigna for the prediction of recurrence/metastasis probability of early-stage breast cancer. Nevertheless, for most other tumor types, effective prognostic or diagnostic biomarkers are greatly needed. OSdream was constructed to screen and validate the key molecules or biomarkers for predicting tumor metastasis or recurrence which may serve clinical translation. Hence, OSdream is a potential and significant prognostic analysis tool for recurrence and metastasis, it will greatly help scientific researchers and clinicians to explore and validate biomarkers of cancer recurrence and metastasis, and identify potential therapeutic targets in pan-cancers. In the future, we will keep collecting data and updating OSdream to gradually improve its performance.

## Data availability

The datasets generated during the current study are available from the corresponding authors upon reasonable request.

## CRediT authorship contribution statement

**Huimin Li:** Conceptualization, Data curation, Writing – original draft, Writing – review & editing. **Qiang Wang:** Software, Visualization. **Yunyi Liu:** Data curation, Validation. **Lin Chen:** Data curation, Validation. **Qiwei Zhao:** Data curation. **Longxiang Xie:** Writing – review & editing. **Lu Zhang:** Writing – review & editing. **Zhongyi Yan:** Writing – review & editing. **Guosen Zhang:** Writing – review & editing. **Yali Han:** Writing – review & editing. **Wan Zhu:** Writing – review & editing. **Xiangqian Guo:** Conceptualization, Writing – review & editing, Supervision.

## Conflict of interests

The authors declared no competing interests.

## Funding

This work was supported by the 10.13039/501100001809National Natural Science Foundation of China (No. U2004136), the Supporting Program for Central Plain Young Top Talents (China) (No. ZYQR201912176), the Program from Academy for Advanced Interdisciplinary Studies of Henan University (No. Y21008L), the Henan Province Scientific and Technology Research Project (China) (No. 232102311102), and the Kaifeng Scientific and Technology Research Project (Henan, China) (No. 2203010).
