# Supplementary material for: OSdream: An online survival and differential analysis tool of recurrence and metastasis of pan-cancers
Source: Genes Dis. 2024 Oct 30;12(4):101446. doi: 10.1016/j.gendis.2024.101446 (PMC11982969; doi:10.1016/j.gendis.2024.101446)
Supplement: Multimedia component 1 [file mmc1.docx]

**Materials and methods**

To construct OSdream, we collected 144 transcriptional profiling datasets with clinical follow-up data of 33 cancer types and 22919 samples from TCGA, GEO databases and literatures. Of these, 50 expression profiling datasets, composed of 12722 samples covering 29 tumor types, have RFS information, while 21 expression profiling datasets, composed of 3505 samples covering 5 tumor types, have MFS information. 50 expression profiling datasets with 13371 samples covering 29 tumor types have normal tissues, primary and recurrent tumor tissues for differential analysis by the DEGs of recurrence module, while 73 expression profiling datasets with 6043 samples covering 21 types of tumor have normal tissues, primary and metastasized tumor tissues for differential analysis by the DEGs of metastasis module.

**Design of OSdream**

To develop OSdream, SQL Server is used for data storage and integration, HTML 5.0 is used to develop the dynamic web interfaces, Java is connected to R by the R package 'R serve' and used to output analysis results, which include survival analysis and differential analysis. OSdream homepage provides four application modules that cater to two functions: prognosis and differential analysis. The four application modules are the RFS (recurrence-free survival) and the MFS (metastasis-free survival) modules for prognosis analysis, the DEGs of recurrence module, and the DEGs of metastasis module for differential analysis of recurrence and metastasis.

**Prognostic analysis of metastasis and recurrence**

To perform the prognosis analysis in OSdream, Kaplan-Meier plot, Cox regression analysis and nomogram are used to analyze metastasis-free survival and recurrence-free survival with R packages including “ggplot2”, “ggpubr”, “magrittr”, “survminer” and “survival”. Survival analysis here is applied to assess the prognostic potency of one gene. Cox regression analysis allows the joint analysis of various prognostic variables either continuous, or discrete, and provides quantitative estimates of patient risk and HR ratio. The nomogram is used to evaluate cancer patient outcome, by simplifying various predictive and prognostic variables into a single numerical estimation.

**Differential analysis of metastasis and recurrence**

For differential analysis, box plots and heatmaps are used to exhibit the differential analysis results of recurrence and metastasis using R packages “pheatmap”, “ggplot2”, “ggpubr”, “ggsci”. Regarding metastasis, the functions of three types of subgroup comparison could be performed, specifically, including comparison of primary tumor *vs* metastatic tumor, primary tumor without metastasis *vs* primary tumor with metastasis, and primary tumor *vs* lymph node metastasis. In addition, OSdream can also output the comparative results between normal tissues, primary tumors and/or metastatic tumors. For recurrence differential analysis, OSdream provides a comparative analysis between tumors that have no history of recurrence and tumors that have experienced recurrence events. OSdream also supports multiple gene differential analysis and outputs expression clustering heatmap, which shows the co-expression pattern of queried genes.
