# Supplementary material for: OSdream: An online survival and differential analysis tool of recurrence and metastasis of pan-cancers
Source: Genes Dis. 2024 Oct 30;12(4):101446. doi: 10.1016/j.gendis.2024.101446 (PMC11982969; doi:10.1016/j.gendis.2024.101446)
Supplement: Multimedia component 2 [file mmc2.doc]

**Supplementary figures**

**
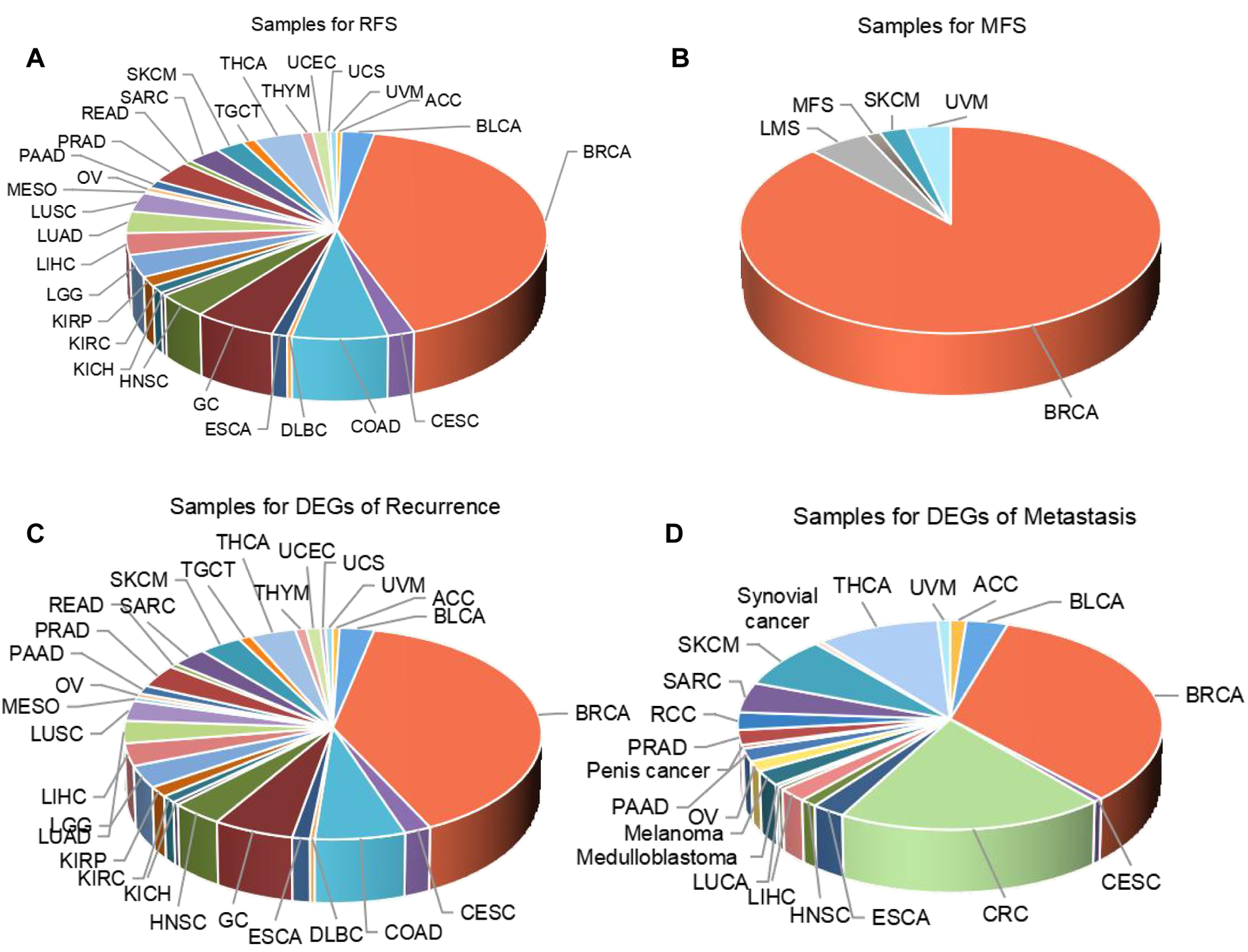
**

**FigureS1** The sample number distribution across different tumor types. The sample number distribution for module RFS (**A**) and module MFS (**B**) for prognostic analysis in OSdream. The sample number distribution for module DEGs of Recurrence (**C**) and module DEGs of Metastasis (**D**) for differential analysis in OSdream.


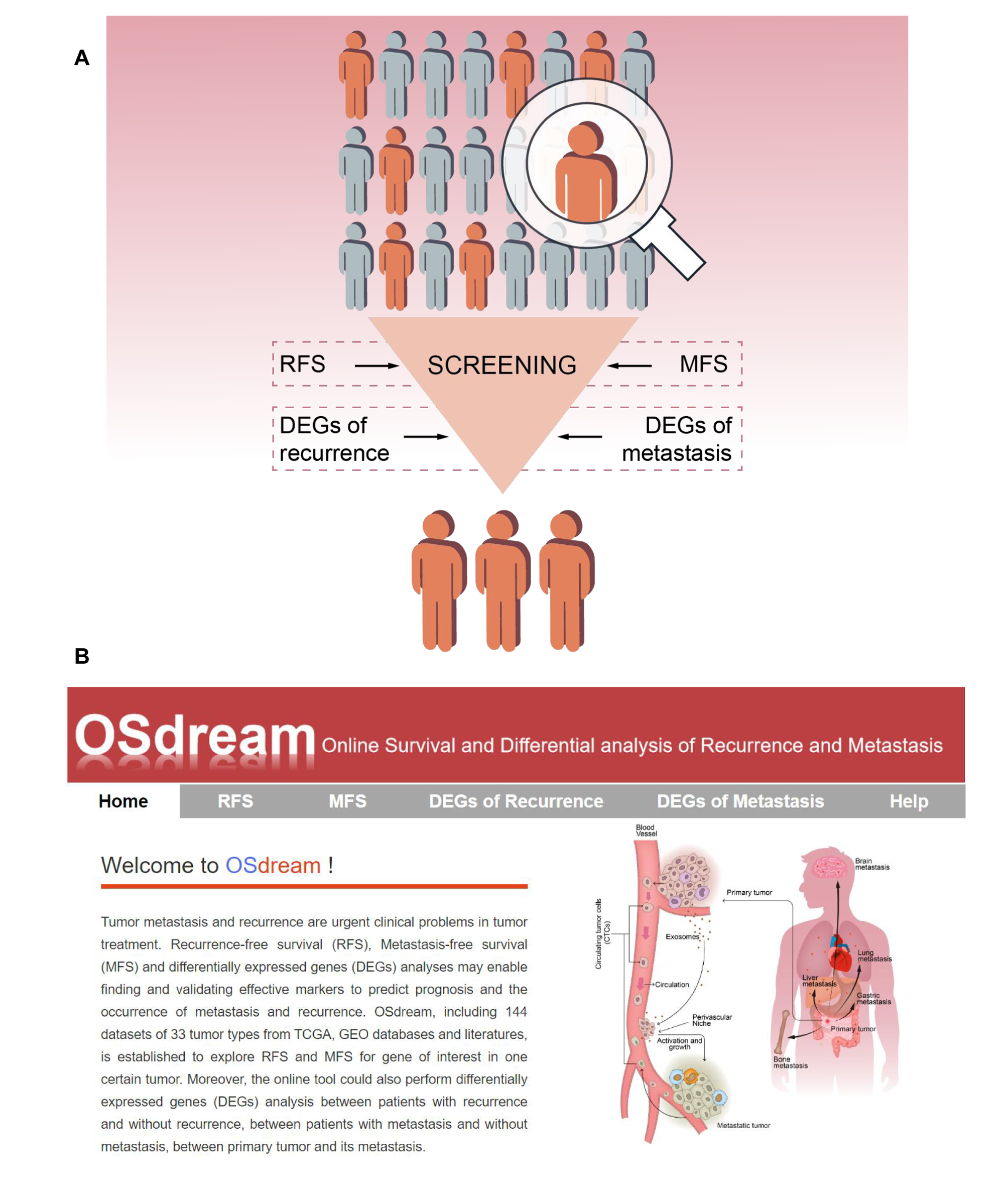


**FigureS2** The design rationale and homepage of OSdream. (**A**) The rationale of four analysis modules in OSdream including RFS, MFS, DEGs of recurrence, and DEGs of metastasis modules. (**B**) The homepage of OSdream.

**
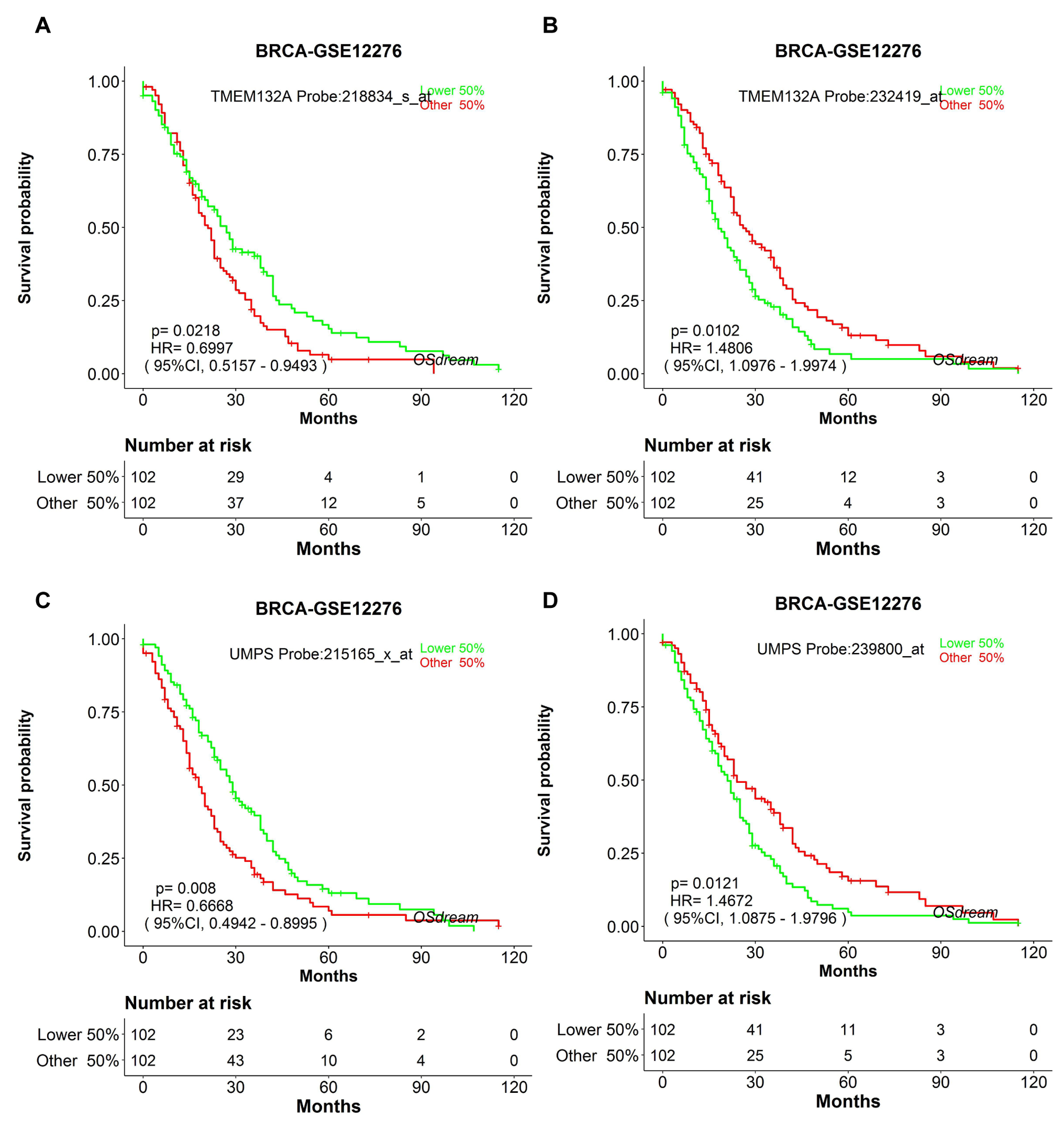
**

**FigureS3:** The evaluation for different probes of one gene in predicting metastasis-free survival in BRCA based on OSdream. (**A**) Probe 218834_s_at of TMEM132A may predict short metastasis-free survival of BRCA patients based on data in GSE12276. (**B**) Probe 232419_at of gene TMEM132A in GSE12276 may predict long metastasis-free survival of BRCA patients. (**C**) Probe 215165_x_at of gene UMPS may predict short metastasis-free survival of BRCA patients based on dataset GSE12276. (**D**) Probe 239800_at of UMPS may predict long metastasis-free survival of BRCA patients based on dataset GSE12276.


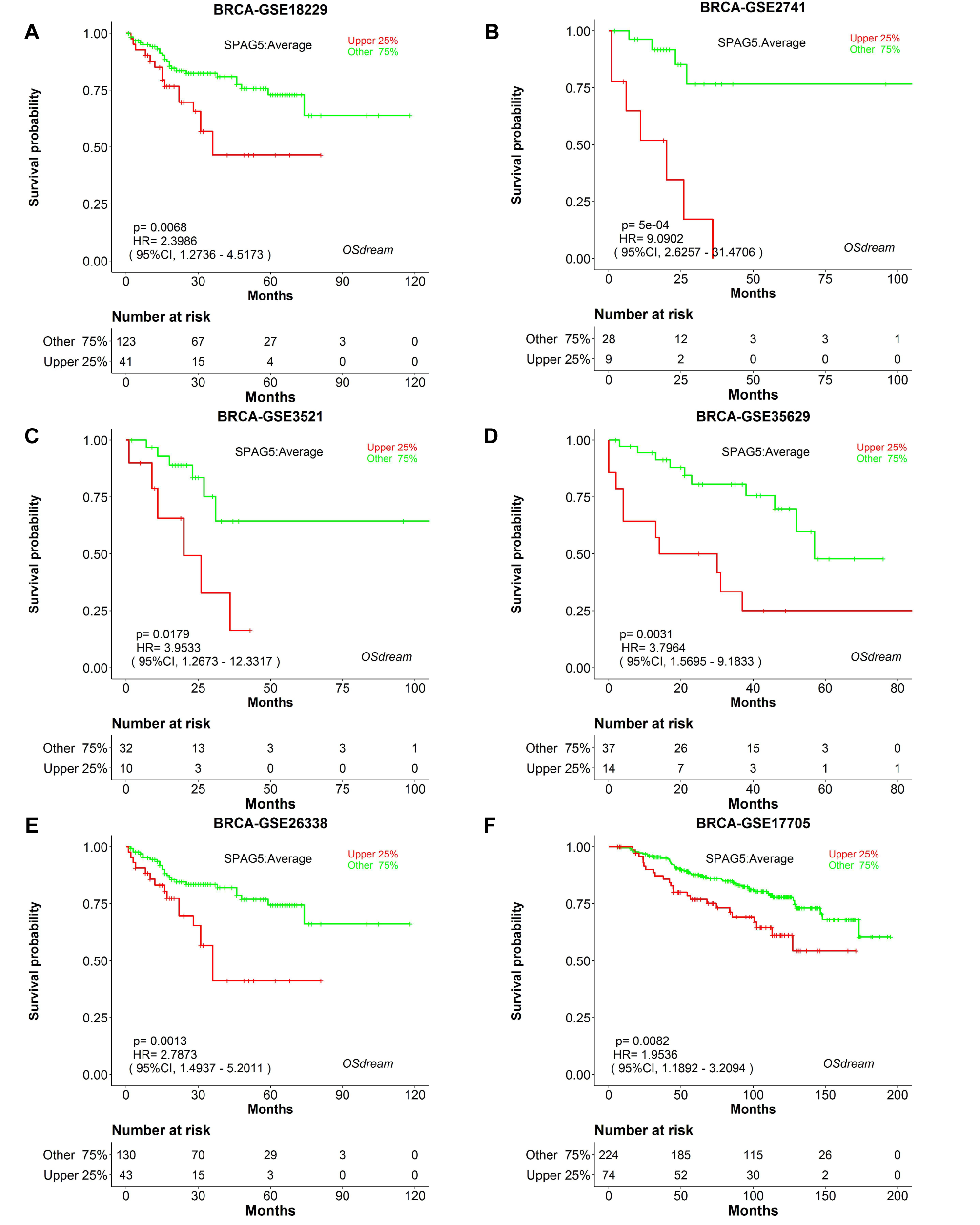


**FigureS4:** **The prognostic role of SPAG5 for predicting recurrence-free survival outcome in BRCA.** SPAG5 predicts short recurrence-free survival in six datasets by RFS module of OSdream, including GSE18229 (**A**), GSE2741 (**B**), GSE3521 (**C**), GSE35629 (**D**), GSE26338 (**E**), GSE17705 (**F**) in BRCA.


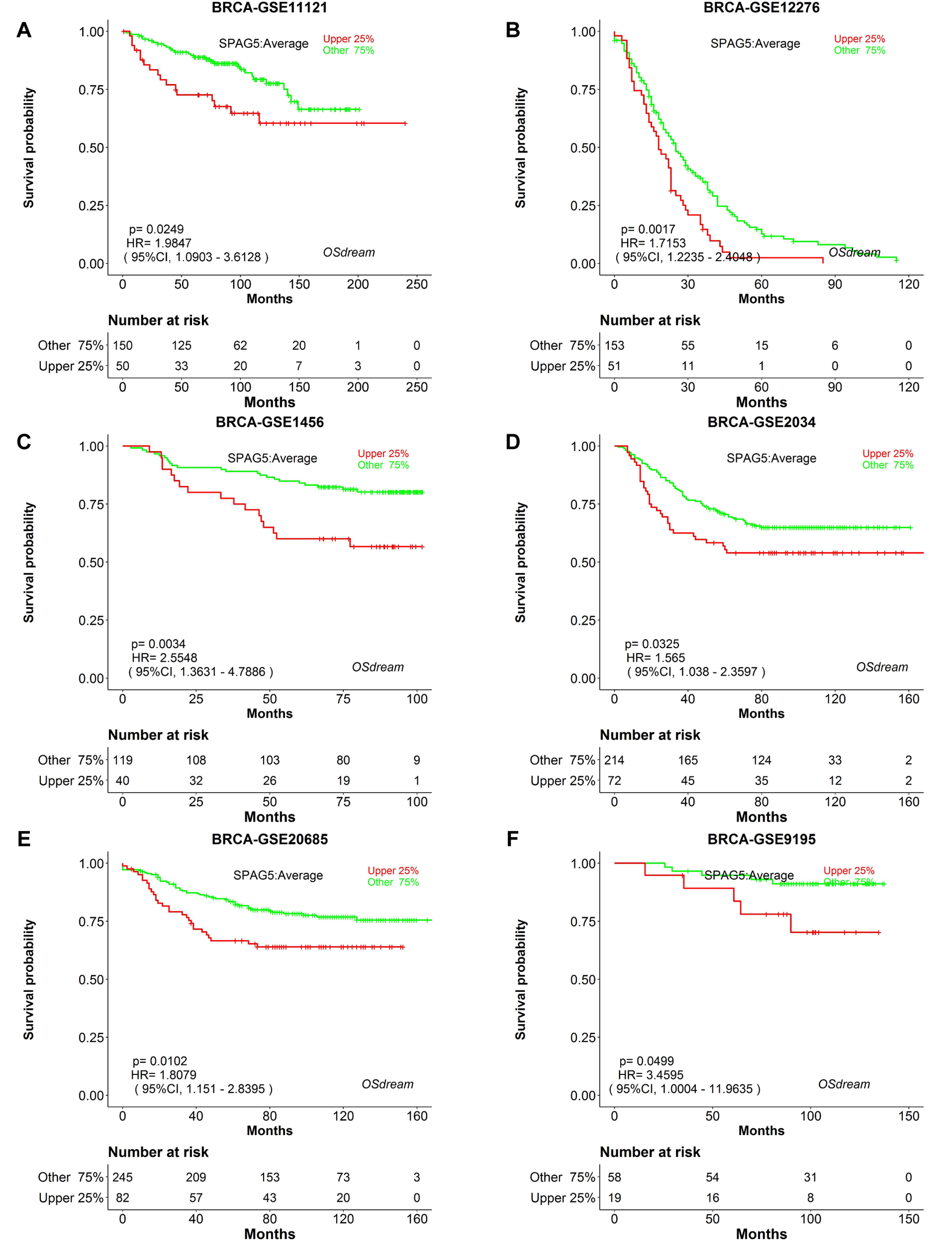


**FigureS5: The prognostic role of SPAG5 for predicting metastasis-free survival outcome in BRCA.** SPAG5 predicts short metastasis-free survival outcome in six datasets by MFS module of OSdream, including GSE11121 (**A**), GSE12276 (**B**), GSE1456 (**C**), GSE2034 (**D**), GSE20685 (**E**), GSE9195 (**F**) in BRCA.


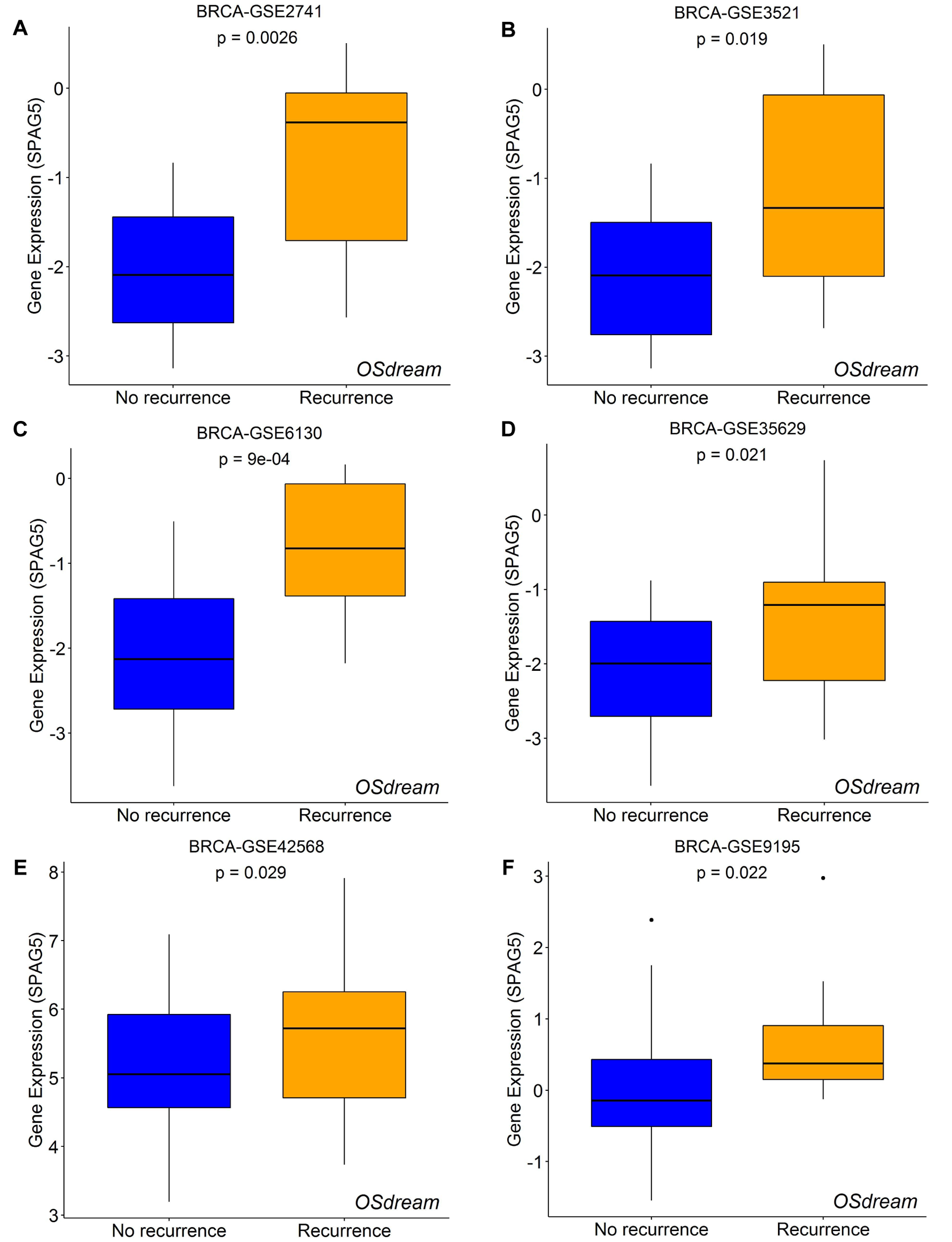


**FigureS6:** **The differential expression analysis of SPAG5 in BRCA using DEGs of recurrence module of OSdream.** SPAG5 is overexpressed in the recurrence group compared to the non-recurrence group in six datasets, including GSE2741 (**A**), GSE3521 (**B**), GSE6130 (**C**), GSE35629 (**D**), GSE42568 (**E**), GSE9195 (**F**) in BRCA.


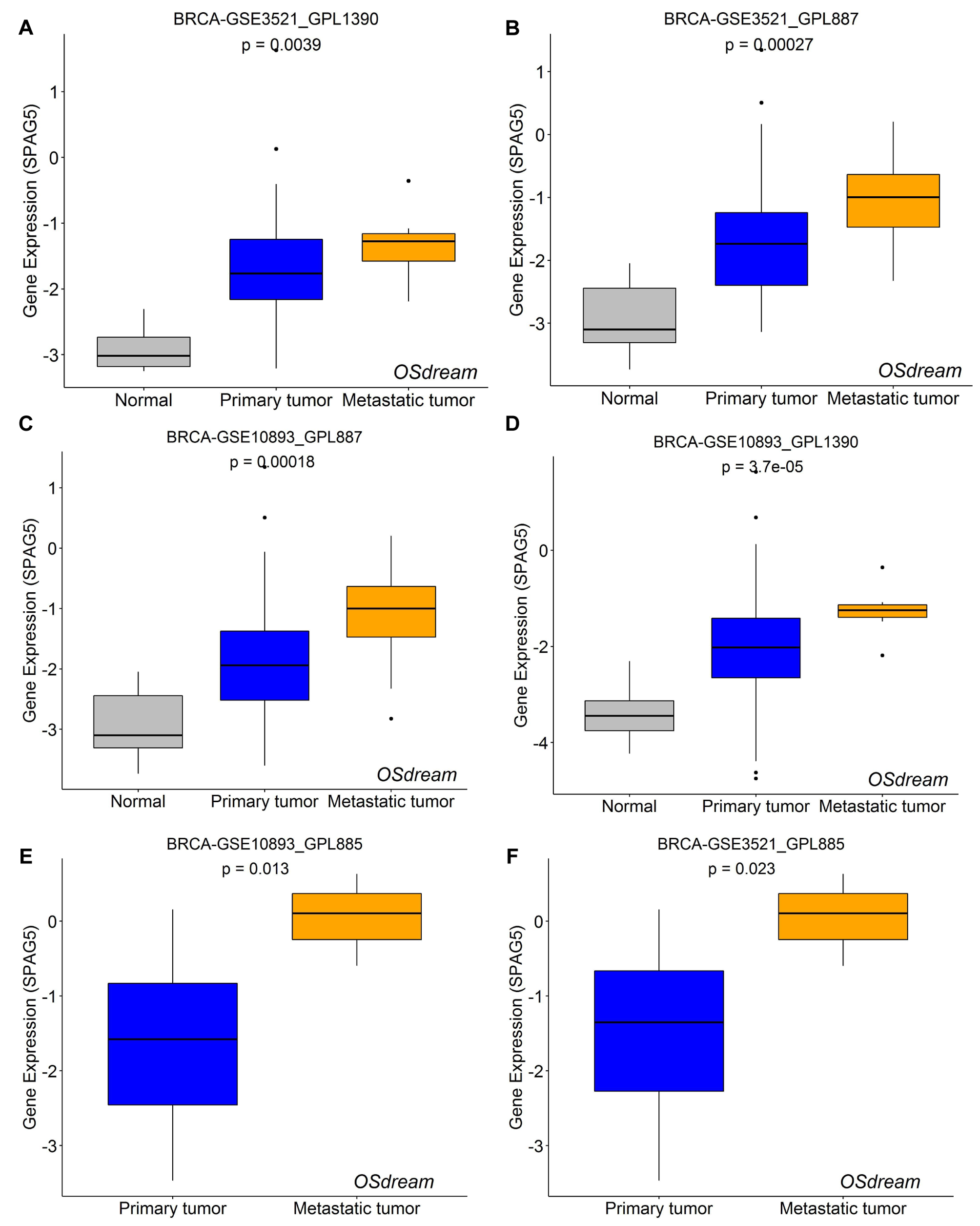


**FigureS7: The differential expression analysis of SPAG5 in BRCA by DEGs of metastasis module in OSdream.** SPAG5 is overexpressed in metastatic tumor tissues compared to primary tumor tissues in six datasets, including GSE3521_GPL1390 (**A**), GSE3521_GPL887 (**B**), GSE10893_GPL887 (**C**), GSE10893_GPL1390 (**D**), GSE10893_GPL885 (**E**), GSE3521_GPL885 (**F**) in BRCA.
