# Supplementary material for: OSdream: An online survival and differential analysis tool of recurrence and metastasis of pan-cancers
Source: Genes Dis. 2024 Oct 30;12(4):101446. doi: 10.1016/j.gendis.2024.101446 (PMC11982969; doi:10.1016/j.gendis.2024.101446)
Supplement: Multimedia component 3 [file mmc3.doc]

**Table S1** The [quantity](javascript:;) and source information of samples used in survival analysis.

| **Survival analysis** | | | | | | |
| --- | --- | --- | --- | --- | --- | --- |
| **RFS** | | |  | **MFS** | | |
| Cancer type | Sample size (n) | Data source |  | Cancer type | Sample size (n) | Data source |
| ACC | 54 | TCGA |  | BRCA | 3086 | GEO |
| BLCA | 336 | TCGA |  | LMS | 173 | GEO |
| BRCA | 5251 | TCGA, GEO |  | MFS | 43 | GEO |
| CESC | 248 | TCGA |  | SKCM | 77 | GEO |
| COAD | 883 | TCGA, GEO |  | UVM | 126 | GEO |
| DLBC | 45 | TCGA |  | Total | 3505 |  |
| ESCA | 143 | TCGA |  |  |  |  |
| GC | 756 | TCGA, GEO |  |  |  |  |
| HNSC | 483 | TCGA |  |  |  |  |
| KICH | 60 | TCGA |  |  |  |  |
| KIRC | 145 | TCGA |  |  |  |  |
| KIRP | 197 | TCGA |  |  |  |  |
| LGG | 440 | TCGA |  |  |  |  |
| LIHC | 428 | TCGA |  |  |  |  |
| LUAD | 429 | TCGA |  |  |  |  |
| LUSC | 386 | TCGA |  |  |  |  |
| MESO | 41 | TCGA |  |  |  |  |
| OV | 83 | TCGA |  |  |  |  |
| PAAD | 156 | TCGA |  |  |  |  |
| PRAD | 436 | TCGA |  |  |  |  |
| READ | 83 | TCGA |  |  |  |  |
| SARC | 351 | TCGA, GEO |  |  |  |  |
| SKCM | 297 | TCGA |  |  |  |  |
| TGCT | 132 | TCGA |  |  |  |  |
| THCA | 498 | TCGA |  |  |  |  |
| THYM | 114 | TCGA |  |  |  |  |
| UCEC | 153 | TCGA |  |  |  |  |
| UCS | 29 | TCGA |  |  |  |  |
| UVM | 65 | TCGA |  |  |  |  |
| Total | 12722 |  |  |  |  |  |

**Table S2.** The [quantity](javascript:;) and source information of samples used in differential expression analysis.

| **Differential analysis** | | | | | | |
| --- | --- | --- | --- | --- | --- | --- |
| **DEGs of Recurrence** | | |  | **DEGs of Metastasis** | | |
| Cancer type | Sample size (n) | Data source |  | Cancer type | Sample size (n) | Data source |
| ACC | 76 | TCGA |  | ACC | 79 | TCGA |
| BLCA | 376 | TCGA |  | BLCA | 201 | TCGA |
| BRCA | 5257 | TCGA, GEO |  | BRCA | 2018 | TCGA, GEO |
| CESC | 263 | TCGA |  | CESC | 40 | GEO |
| COAD | 887 | TCGA, GEO |  | CRC | 1168 | GEO |
| DLBC | 47 | TCGA |  | ESCA | 146 | TCGA |
| ESCA | 174 | TCGA |  | HNSC | 73 | GEO |
| GC | 791 | TCGA, GEO |  | LIHC | 129 | GEO |
| HNSC | 505 | TCGA, GEO |  | LUCA | 28 | GEO |
| KICH | 63 | TCGA |  | Medulloblastoma | 22 | GEO |
| KIRC | 147 | TCGA |  | Melanoma | 139 | GEO |
| KIRP | 211 | TCGA |  | OV | 97 | GEO |
| LGG | 457 | TCGA |  | PAAD | 121 | GEO |
| LIHC | 460 | TCGA, GEO |  | Penis cancer | 33 | GEO |
| LUAD | 456 | TCGA |  | PRAD | 138 | GEO |
| LUSC | 414 | TCGA |  | RCC | 163 | GEO |
| MESO | 80 | TCGA |  | SARC | 282 | TCGA, GEO |
| OV | 83 | TCGA |  | SKCM | 473 | TCGA |
| PAAD | 170 | TCGA |  | Synovial cancer | 34 | GEO |
| PRAD | 470 | TCGA |  | THCA | 599 | TCGA, GEO |
| READ | 85 | TCGA |  | UVM | 60 | TCGA, GEO |
| SARC | 388 | TCGA, GEO |  | Total | 6043 |  |
| SKCM | 466 | TCGA |  |  |  |  |
| TGCT | 135 | TCGA |  |  |  |  |
| THCA | 501 | TCGA |  |  |  |  |
| THYM | 120 | TCGA |  |  |  |  |
| UCEC | 155 | TCGA |  |  |  |  |
| UCS | 55 | TCGA |  |  |  |  |
| UVM | 79 | TCGA |  |  |  |  |
| Total | 13371 |  |  |  |  |  |

**Table S3.** The specific group type and the sample size in each group in DEGs of metastasis module.

| **DEGs of metastasis** | | | | | |
| --- | --- | --- | --- | --- | --- |
| **Cancer type** | **Datasets** | **Metastasis type** | **Primary (n)** | **Metastasis (n)** | **Normal (n)** |
| **ACC** | TCGA | Primary tumor without metastasis Primary tumor with metastasis | 74 | 5 |  |
| **BLCA** | TCGA | Primary tumor without metastasis Primary tumor with metastasis | 146 | 55 |  |
| **BRCA** | GSE3521-  GPL885 | Primary-metastasis | 9 | 3 |  |
|  | GSE3521-  GPL887 | Primary-metastasis | 58 | 16 | 5 |
|  | GSE3521-  GPL1390 | Primary-metastasis | 80 | 8 | 4 |
|  | GSE5327 | Primary tumor without metastasis Primary tumor with metastasis | 47 | 11 |  |
|  | GSE9893 | Primary tumor without metastasis Primary tumor with metastasis | 107 | 48 |  |
|  | GSE10893-  GPL885 | Primary-metastasis | 11 | 3 |  |
|  | GSE10893-  GPL887 | Primary-metastasis | 67 | 16 | 5 |
|  | GSE10893-GPL1390 | Primary-metastasis | 155 | 7 | 8 |
|  | GSE14682 | Primary-metastasis | 14 | 46 |  |
|  | GSE30480 | Primary-metastasis | 14 | 6 |  |
|  | GSE38057 | Primary tumor without metastasis Primary tumor with metastasis | 46 | 41 |  |
|  | GSE46563 | Primary tumor without metastasis Primary tumor with metastasis | 71 | 23 |  |
|  | GSE46928 | Primary tumor without metastasis Primary tumor with metastasis | 41 | 11 |  |
|  | GSE76124 | Primary tumor without metastasis Primary tumor with metastasis | 148 | 33 |  |
|  | GSE76714 | Primary tumor without metastasis Primary tumor with metastasis | 48 | 23 |  |
|  | TCGA | Primary tumor without metastasis Primary tumor with metastasis | 771 | 14 |  |
| **CESC** | GSE7410 | Primary tumor without metastasis Primary tumor with metastasis | 21 | 19 |  |
| **CRC** | GSE14297 | Primary-metastasis | 18 | 18 |  |
|  | GSE18462 | Primary-metastasis | 2 | 2 | 2 |
|  | GSE49355 | Primary-metastasis | 20 | 19 |  |
|  | GSE62321 | Primary-metastasis | 20 | 19 |  |
|  | GSE68468 | Primary-metastasis | 195 | 68 | 103 |
|  | GSE18105 | Primary-metastasis | 67 | 26 |  |
|  | GSE64256 | Primary-metastasis | 99 | 26 |  |
|  | GSE9348 | Primary-metastasis | 52 | 18 |  |
|  | GSE87211 | Primary-metastasis | 336 | 21 |  |
|  | GSE26571 | Primary-metastasis | 12 | 11 |  |
|  | GSE40367 | Primary-metastasis (matched) | 7 | 7 |  |
| **ESCA** | TCGA | Primary tumor without metastasis Primary tumor with metastasis | 94 | 52 |  |
| **HNSC** | GSE1722-  GPL96 | Primary-Lymph node metastasis | 6 | 2 | 4 |
|  | GSE1722-  GPL1384 | Primary-Lymph node metastasis | 6 | 2 | 4 |
|  | GSE9349 | Primary-metastasis | 11 | 11 |  |
|  | GSE2280 | Primary-Lymph node metastasis | 22 | 5 |  |
| **LIHC** | GSE45114 | Primary-metastasis | 11 | 13 |  |
|  | GSE28248 | Primary-lymph node metastasis | 20 | 20 |  |
|  | GSE27635 | Primary-metastasis | 19 | 24 |  |
|  | GSE40367 | Primary-metastasis | 6 | 16 |  |
| **LUCA** | GSE1987 | Primary-metastasis | 25 | 3 |  |
| **Medulloblastoma** | GSE63668 | Primary-metastasis | 9 | 13 |  |
| **Melanoma** | GSE15605 | Primary-metastasis | 46 | 12 | 16 |
|  | GSE62837 | Primary-metastasis | 3 | 2 |  |
|  | GSE7553 | Primary melanoma without metastasis Primary melanoma with metastasis | 16 | 40 | 4 |
| **OV** | GSE73091 | Primary-metastasis(mathched) LGSOC | 3 | 3 |  |
|  | GSE30587 | Primary-Omental metastasis (mathched) | 9 | 9 |  |
|  | GSE102180 | Primary-metastasis EOC | 44 | 29 |  |
| **PAAD** | GSE19280 | Primary-Liver metastasis | 4 | 5 | 3 |
|  | GSE19279 | Primary-Liver metastasis | 4 | 5 | 3 |
|  | GSE73338 | Primary-metastasis | 81 | 7 | 9 |
| **Penis cancer** | GSE85730 | Primary tumor without metastasis Primary tumor with metastasis | 17 | 8 | 8 |
| **PRAD** | GSE6752 | Primary-metastasis | 10 | 21 |  |
|  | GSE32269 | Primary-metastasis | 22 | 29 |  |
|  | GSE74367 | Primary-metastasis | 11 | 45 |  |
| **RCC** | GSE12606 | Primary-metastasis | 3 | 3 |  |
|  | GSE23629 | Primary-metastasis | 16 | 16 |  |
|  | GSE85258 | Primary-metastasis | 15 | 16 |  |
|  | GSE22541 | Primary-metastasis | 24 | 44 |  |
|  | GSE31232 | Primary-metastasis | 6 | 2 |  |
|  | GSE31610 | Primary-metastasis | 7 | 2 |  |
|  | GSE47352 | Primary-metastasis | 5 | 4 |  |
| **SARC** | GSE14359 | Primary-lung metastasis | 10 | 8 | 2 |
|  | GSE32981 | Primary-metastasis | 12 | 11 |  |
|  | GSE36001 | Primary-metastasis | 7 | 3 |  |
|  | GSE49327 | Primary-metastasis | 5 | 8 |  |
|  | GSE12102 | Primary-metastasis | 30 | 7 |  |
|  | TCGA | Primary tumor without metastasis Primary tumor with metastasis | 120 | 59 |  |
| **SKCM** | TCGA | Primary tumor without metastasis Primary tumor with metastasis | 402 | 71 |  |
| **Synovial cancer** | GSE40018 | Primary melanoma without metastasis Primary melanoma with metastasis | 17 | 17 |  |
| **THCA** | GSE60542 | Primary-metastasis | 33 | 23 | 30 |
|  | TCGA | Primary tumor without metastasis Primary tumor with metastasis | 500 | 13 |  |
| **UVM** | GSE73652 | Primary tumor without metastasis Primary tumor with metastasis | 8 | 5 |  |
|  | TCGA | Primary tumor without metastasis Primary tumor with metastasis | 44 | 3 |  |
| **Total** |  |  | 4519 | 1314 | 210 |

**Table S4.** The validation of four modules in OSdream with prior biomarkers.

| **Gene symbol** | **Module** | **Cancer type** | **Datasets** |
| --- | --- | --- | --- |
| POR | RFS | BRCA | GSE26338, GSE2741 |
| HSF4 | RFS | COAD | TCGA |
| DAAM1 | MFS | BRCA | GSE11121, GSE20685 |
| CDK6 | MFS | MFS | GSE71118 |
| HE4 | DEGs of Recurrence | OV | TCGA |
| POR | DEGs of Recurrence | BRCA | GSE10894, GSE18229, GSE26338, GSE2741, GSE9195 |
| MUC1 | DEGs of Metastasis | BRCA | GSE9893, GSE14682 |
| AFP | DEGs of Metastasis | PRAD | GSE6752, GSE74367 |
